# Memory-directed acupuncture as a neuromodulatory treatment for PTSD: Theory, clinical model and case studies

**DOI:** 10.1038/s41398-022-01876-3

**Published:** 2022-03-17

**Authors:** Amir Assouline, Avi Mendelsohn, Alon Reshef

**Affiliations:** 1 Sagol Department of Neurobiology, University of Haifa, Haifa, Israel; 2 Institute for Information Processing and Decision Making, University of Haifa, Haifa, Israel; 3 Ha’Emek Medical Center, Department of Psychiatry, Afula, Israel

**Keywords:** Psychiatric disorders, Learning and memory

## Abstract

Posttraumatic stress disorder (PTSD) poses an ongoing challenge to society, to health systems, and to the trauma victims themselves. Today PTSD is often considered an incurable chronic problem that lacks effective treatment. While PTSD is closely related to memory, it also affects many physiological systems. PTSD is usually treated with medications and psychotherapy with moderate success, leaving a substantial proportion of patients with enduring distress and disability. Therefore, a search for better treatment options is vital. In this paper, we propose a model in which a conversation-based technique is integrated with bodily manipulation through acupuncture. This approach first emerged in clinical experience showing intriguing results from treating PTSD patients using acupuncture as a main strategy. Its theoretical foundations derive from the clinic and rely on contemporary neuroscience’s understanding of memory consolidation and reconsolidation processes. Research shows that acupuncture can have potentially positive effects at three levels: (a) achieving a balance between sympathetic and parasympathetic neural activity; (b) reducing activation in the limbic system, hence inducing a calming effect; (c) reshaping the functional connectivity map within important and relevant cortical regions that encompass the default-mode network. We suggest that coupling traumatic memory retrieval leading to reconsolidation, combined with acupuncture, offers considerable potential for positive clinical improvement in patients with PTSD. This may explain the positive results of the described case studies and can pave the path for future advances in research and treatment in this field.

## Introduction

Posttraumatic stress disorder (PTSD) as defined by the DSM-5 results from exposure to an extreme event and poses a threat to bodily integrity. PTSD can be brought on by direct experience of serious injury or sexual violence, by witnessing or having knowledge of violent injury or threat of death to a family member or friend, or by exposure to dreadful or frightening incidents at work (e.g., among investigators, police officers, firefighters, rescue and recovery personnel) [1]. A key component of posttraumatic stress disorder is traumatic memory, whether overt or latent, which exerts a tremendous impact on physiological and mental capacities and consequently on the individual’s ability to function appropriately in the present [2].

Despite the vast personal and economic burden of PTSD, existing treatments are limited. Some treatment methods attempt to treat the disorder’s symptoms, among them eye-movement desensitization and reprocessing (EMDR), prolonged exposure (PE), and pharmacological treatment. Yet overall, psychotherapy is thought to offer the greatest efficacy in treating PTSD [3]. Some studies show that beyond the cognitive level, successful psychotherapy for PTSD may alter related biomarkers, such as dysregulations along the hypothalamus–pituitary–adrenal (HPA)-axis [4], and that cognitive-behavioral treatment can affect gene expression and hippocampal volume [5]. Nevertheless, the overall effectiveness of PTSD treatments is still highly limited, as is evident in the high percentage of treatment-resistant patients. For instance, in psychotherapy-based studies, more than 30% of patients who completed a full course of treatment continued to meet the criteria for PTSD [6].

Individuals with PTSD may experience intrusive memories, avoidance, personality changes, and negative attitudes and beliefs regarding the self and the world. In addition, PTSD has high co-morbidity with anxiety and depression. What sets posttraumatic stress disorder apart from other disorders is that PTSD is associated with specific, highly emotional memories [7, 8]. Recurring symptoms, such as flashbacks, intrusive memories, and trauma-related nightmares, are considered hallmark symptoms of PTSD [9]. Although the source of PTSD lies in the past, the perception of present reality in posttraumatic individuals may be altered as well, such that relatively minor threats are exaggerated, the ability to cope with the burden of reality is underestimated, and the individual adopts a view of the world as a threatening and intimidating place. Cognitive therapy focuses mainly on these aspects [10]. In addition, over time a broader cognitive impairment may develop, which among other things is manifested in reduced concentration and memory capacities [11].

PTSD has several noteworthy physiological and pathophysiological manifestations. One is hyperarousal, as manifested in increased heart rate and skin conductance in response to trauma-related cues. Another is an imbalance in the sympathetic-parasympathetic nervous system that is not adequately regulated by the HPA axis, as manifested in unregulated (increased or decreased) secretion of stress hormones [12], including cortisol and other glucocorticoids. Such an imbalance can lead to further chronic illnesses, among them metabolic syndrome, exaggerated inflammation processes, shortened telomere lengths, and more [3, 13]. Though psychotherapy has been found to alter some physiological biomarkers of PTSD, it does not appear to encompass the whole illness. PTSD is not merely a cognitive impairment or a neural abnormality, but rather a systematic illness that entails both cognitive and physiological burdens [13]. Thus, in order to treat PTSD, the cognitive and physiological axes must be merged into a united framework [2, 13, 14].

In the present paper, we sought to address this demand for a united framework for treating PTSD. We suggest that the application of acupuncture—a physiological manipulation—together with memory retrieval may significantly affect the reconsolidation process through the demonstrated effect of acupuncture on neural systems. Thus, this combination may significantly alter the traumatic event memory representation and the resultant PTSD symptoms. Our clinical model is based on neuroscientific research that sees acupuncture as a neuromodulator as well as a means of affecting the neural underpinnings of memory processes. This model is supported by clinical cases in which this approach yielded tremendous changes in PTSD symptoms and general well-being. Two such clinical case studies are described here.

From a broader perspective, the intersection between the physiological and cognitive axes is one of the cornerstones of Chinese medicine [15] and can be applied in treating PTSD [2, 14]. For treatment to be effective, these axes must meet, since the dissociation and separation between them is a key feature of the pathology [13]. We hope that laying the theoretical foundations and scientific rationale of this approach will pave the path for further research that may bring us a step closer to an effective treatment for PTSD.

## Memory processes, consolidation, and reconsolidation

By definition, PTSD stems from past events [16] and is therefore inherently tied to memory representations (engrams) and memory-related processes. Encoded events undergo a maturation process, starting off fragile and susceptible to interference and slowly maturing into more solid representations throughout the postulated consolidation process [17–20]. Whereas newly encoded information is sensitive to change [19], post-consolidation memories are more immune to manipulation. The process of consolidation takes place primarily during sleep [21], although initial consolidation can begin immediately post-encoding [22]. During consolidation, new synaptic connections are formed [23, 24]. For instance, protein synthesis in the amygdala is required for fear conditioning to occur [25]. Recent advances in memory research indicate that consolidation does not terminate upon the transfer of information from short-term to long-term memory, but rather may undergo dynamic changes across multiple timescales [17, 19, 20, 26].

Indeed, animal studies show that the administration of protein synthesis inhibitors such as anisomycin during the labile phase, in temporal proximity to the original encoding event, prevents consolidation and moderates posttraumatic consequences in rats [27]. Yet not only does the initial establishment of memory following the first experience or learning require protein synthesis, but in certain cases *reconsolidation* following memory retrieval requires protein synthesis as well [28, 29]. During reconsolidation, stored information is rendered labile after being retrieved, and pharmacological manipulations at this stage result in an inability to retrieve the memories at later times, suggesting that they are either erased or persistently inhibited [30]. In fear conditioning, for instance, protein synthesis is required after each retrieval [31]. The possibility of retroactive intervention in an existing memory that has already been consolidated opens a wide range of possible interventions for traumatic memory therapy, though some aspects of this process are still debated [18, 32]. Yet pharmacological interventions based on the administration of protein synthesis inhibitors (e.g., anisomycin) are not possible in humans. Hence, alternative treatments that can induce memory reconsolidation in humans are needed [29, 33]. This type of intervention can be of major significance in treating PTSD. The brain mechanism that facilitates reconsolidation may also underlie the changes that follow psychotherapy, in which the traumatic event is retrieved and reprocessed in a protected environment, for example in prolonged exposure [33] where the patient is re-exposed to a situation reminiscent of the traumatic event. This method has been found to be effective in treating PTSD.

Some researchers have suggested pharmacological intervention using propranolol to enhance the therapeutic effect of psychotherapy in boosting memory destabilization [33]. Animal research shows that this coupling does in fact accelerate the extinction of fear conditioning during reconsolidation, although human research is still deficient and controversial [34, 35]. As mentioned, post-trauma theorists and clinicians emphasize that traumatic memory is not only embedded in cerebral memory systems but involves physiological experience as well [2]. The attempt to bring about memory reorganization through a conversation that naturally addresses only the cognitive plane does not seem to delve into the somatic experience in which traumatic memory is anchored. Indeed, some body-mind therapies, such as somatic experiencing [36], are designed to address exactly that level.

In summary, the basic paradigm: memory retrieval—destabilized phase; external intervention—reprocessing/reorganization; and reconsolidation, is a promising direction. Nevertheless, the manipulation must be adjusted to the intensity at which the traumatic memory is enacted.

Our clinical experience in a private clinic and a hospital psychiatric clinic shows that a combination of memory retrieval by means of prior conversation and subsequent acupuncture treatment for reconsolidation results in significant clinical improvement. We suggest that the physiological and mental effects of acupuncture may serve as an interface for assimilating the cognitive changes occurring during reconsolidation into the physical systems in which the traumatic memory is anchored.

## The biology of acupuncture

Chinese medicine is based on a broad corpus of anatomical, physiological, and clinical knowledge regarding human physiology and psychology in both health and disease. It encompasses comprehensive philosophical conceptions, as well as physiological and pathophysiological theory regarding the cause and treatment of diseases and therapeutic practice rooted in clinical and philosophical theories [15]. Chinese medicine treatment typically relies on acupuncture and herbal medicine. Acupuncture refers to a network of longitudinal meridians and transverse pathways on the surface of the skin that are functionally parallel to internal organs and their interactions. Acupuncture points are scattered along the meridians and are usually designated according to their “geographical” location and their clinical influence. An extensive body of clinical research discusses the effects of acupuncture points [37] and attempts to compare meridians to known neural pathways [38]. To date, however, there is no general model that directly explains how the therapeutic influence of the acupoints corresponds with their location on the surface of the skin [39]. Nevertheless, although the biological rationale for the morphology of the meridians is not yet clear, today the existence of these meridians and their basic clinical and physiological impact are broadly accepted, and this acceptance is growing as research progresses [39]. The effect of acupuncture on physiological systems has been well established through hundreds of randomized controlled trials (RCT) conducted in recent decades to explore the effectiveness of acupuncture. These studies can be divided into two types: those that compare acupuncture to treatment or lack of treatment (pragmatic trials) and those that compare acupuncture to placebo (efficacy trials) [40]. In addition, some studies seek to characterize the mechanism underlying the apparent effects of acupuncture [41, 42]. Moreover, an extensive body of theoretical literature attempts to outline probable models that explain acupuncture’s effectiveness [43]. This literature includes basic scientific research conducted without any deliberate connection to acupuncture. One example of such research examines the mechanism underlying the production and regulation of inflammation. The body’s inflammatory and anti-inflammatory responses must be balanced and accurate. A deficient inflammatory response can lead to infections and even cancer, while an excessive inflammatory response over time can be more dangerous than the cause of the inflammation itself, manifesting in diseases such as Crohn’s, rheumatoid arthritis, atherosclerosis and more [44]. Inflammatory regulation is mediated among other things by the vagus nerve. Research shows that acupuncture increases vagal modulation and can therefore reduce excess inflammatory activity over time [45]. Tracey [44] describes the relationship between the nervous system and inflammatory processes: Sensory nerves carry inflammation-related information to the hypothalamus, which responds by initiating an anti-inflammatory process mediated by the autonomic nervous system (ANS). More specifically, an inflammatory response is triggered by activation of TNF (tumor necrosis factor) and other cytokines. Parasympathetic activity, on the other hand, is mediated by the vagus nerve, which activates nicotinic macrophage receptors [45], resulting in cholinergic activity which in turn regulates inflammatory cytokines [44].

Similarly, the actions of four neural pathways that terminate in the periventricular nucleus (PVN) of the hypothalamus stimulate or inhibit inflammatory activity in the body. One of these is a distinct pathway whose effect can be tracked by PET and MRI scanning. This pathway begins in the periventricular nuclei and arcuate nucleus in the hypothalamus and projects through the periaqueductal gray to the Raphe nuclei and the dorsal horn of the spinal cord [46]. The above mechanisms or pathways demonstrate how nerve stimulation can lead to the initiation or silencing of an inflammatory process, thus offering a model to explain acupuncture as a nerve stimulus resulting in a brain response that is mediated by the autonomic nervous system and affects internal physiological processes such as inflammation. These mechanisms also demonstrate how basic science can support our understanding and conceptualization of acupuncture. Inflammation mechanisms are not specifically within the scope of this article, which focuses on memory retrieval coupled with acupuncture. Nevertheless, these mechanisms are relevant to PTSD in that multiple studies have demonstrated immune activation in PTSD that is consistent with inflammation. PTSD patients experience increased inflammatory reaction, probably due to consistent impairment in HPA-axis regulation [3, 12]. As described in the next section, acupuncture has been shown to influence HPA-axis regulation.

## Acupuncture and the brain

In recent years, many new means for exploring brain activity in health and illness have been developed. Specifically, the use of fMRI has significantly advanced the understanding of dynamic brain activity. Hence, a great deal of fMRI research has been devoted to uncovering the biological mechanism and neural underpinnings of acupuncture [43].

Taken together with the PTSD features and memory mechanisms surveyed above, a survey of current neuroimaging research on the neurobiological underpinnings of acupuncture suggests three avenues through which acupuncture may modulate physiological systems that correspond with PTSD and may enhance reconsolidation when combined with memory retrieval.

### Sympathetic-parasympathetic balance

Several studies have found that acupuncture can induce an increase in cerebral connectivity and oxygenated blood in the periaqueductal gray area [47], an area rich in opiate receptors. Activity in this area can result in analgesia through the activation of inhibitory pathways that inhibit the processing and perception of pain, a process that may explain part of the established analgesic effect of acupuncture [43]. Moreover, the periaqueductal gray area demonstrates dense reciprocal connectivity with the limbic system and plays a significant role in PTSD, especially in dissociative states [14]. Studies demonstrate that acupuncture can regulate the autonomic nervous system, improve sympathetic/parasympathetic balance and affect parts of the limbic system and cortical regions associated with memory [48].

### Acupuncture effects on the limbic system

#### The amygdala

Neuroimaging findings in posttraumatic stress disorder indicate that elevated amygdala activity [49] can predict predisposition to PTSD [50] and is crucial to fear memory extinction in animals [31]. The most consistent functional abnormality found in human PTSD studies is a hyperactive amygdala in response to emotional stimuli, whether trauma-specific or not [51]. A meta-analysis of resting-state functional MRI revealed consistent hyperactivity in the amygdala, parahippocampus and anterior cingulate cortex in PTSD patients compared to healthy controls [49]. Some neuromodulators proved effective in reducing amygdala hyperactivity to some extent. For instance, deep brain stimulation (DBS) was found to be superior to medical treatment in rat models of PTSD [52]. Moreover, functional MRI showed that vagal nerve stimulation (VNS) activates the nucleus tractus solitarius (NTS) and locus coeruleus in the brainstem and leads to a reduction in the BOLD signal in limbic networks, specifically the amygdala, hippocampus, posterior cingulate gyrus, parahippocampal gyrus and middle and superior temporal gyrus [53, 54]. Accordingly, most studies that examine transcranial direct current stimulation (tDCS) for PTSD target the DLPFC or the VMPFC. This, in turn, may modulate the response of the amygdala (and hippocampus, see below), such that PTSD patients who underwent tDCS after (but not during) extinction of fear memory exhibited a decreased response to the negative stimulus during recall. This suggests the existence of a small period of time after extinction learning when memory is malleable, such that tDCS applied during this window may augment consolidation of extinction memory [42]. During these studies, neuromodulation was detached from real traumatic memory retrieval.

Electroconvulsive therapy (ECT) is thought to interfere with memory reconsolidation when applied during retrieval, yet there have been few case studies and no prospective controlled studies of ECT for PTSD [54]. To provide a framework for understanding acupuncture from the neurobiological perspective, Napadow [55] suggests seeing acupuncture as a neuromodulator. Indeed, Napadow found that acupuncture results in decreased activation of the amygdala following verum more than sham acupoints during pain treatment [56]. Moreover, repeated acupuncture treatments have been found to modulate amygdala resting-state functional connectivity by enhancing the amygdala’s functional connectivity with the anterior cingulate cortex and the parahippocampus, while the strength of this connectivity was positively associated with corresponding clinical improvement [57]. Specifically, studies have shown deactivation in limbic and pre-limbic areas, including the amygdala, following acupuncture of LIV3 and LIV2—two known acupoints in the medial-dorsal part of the foot [58]. In addition, acupoint PC6 was found to affect the functional connectivity of the amygdala and the default-mode network (DMN), along with the anterior cingulate cortex (ACC), which also functions abnormally in PTSD following verum but not sham acupuncture. Increased connectivity of the amygdala and the DMN also correlated with an increase in parasympathetic-related HRV metric [48]. A fMRI study demonstrated that St 36, a central point distal to the anterior tubercle of the tibia, modulated neural activity at multiple levels of the cerebro-cerebellar and limbic systems, including the amygdala and the hippocampus [58, 59]. Punctuation of ST36 also yielded greater coherence within the amygdala-related network, according to fMRI functional connectivity analysis. Taken together, these findings offer promising potential for positive neuromodulation in PTSD patients based on acupuncture effects on the amygdala combined with other calming effects. Acupuncture may be more valuable if administered directly post-retrieval during the reconsolidation phase, as we suggest in this clinical model.

#### The hippocampus

The hippocampus is essential to memory consolidation and reconsolidation processes, and is part of the neurocircuitry of (contextual) fear conditioning along with the amygdala and prefrontal cortex [32, 60]. Resting-state functional connectivity in PTSD patients unveil decreased connectivity between mPFC, OFC and (para) hippocampus [49] and increased connectivity with the insula as part of a disrupted equilibrium between the salience and default-mode networks [61]. Studies have shown decreased connectivity in resting state between the nodes of the DMN, including the hippocampus, in PTSD patients compared to controls. Hippocampal activation during a virtual Morris water task under functional neuroimaging was inversely correlated with PTSD symptom severity [62]. On the other hand, studies have demonstrated hyperreaction of the hippocampus to unconditioned aversive stimulus in PTSD patients [63].

According to some accounts, resistant PTSD is derived from an impairment in extinction learning of fear memory. The hippocampus plays a crucial role in the recall of fear extinction through its direct and indirect (via the mPFC) influences on the amygdala [64]. Preclinical work suggests that high frequency stimulation delivered at particular timeframes to the hippocampus, amygdala and prefrontal cortex may improve fear extinction and anxiety-like behavior in rodents [65]. From a different perspective, studies have shown that there is stronger connectivity between the hippocampus and pain-related cerebral regions, such as the periacqueductal gray (PAG), in cases of chronic pain [66]. and multivariate resting-state functional connectivity predicts responses to real and sham acupuncture treatment in chronic low back pain [67]. The coupling of pain and memory is a fundamental aspect in PTSD as well, though it is physical pain that is explored in chronic pain. Research has shown that acupuncture can affect hippocampal activation and functional connectivity with other brain regions. For instance, repeated verum (real acupoints) but not placebo acupuncture normalizes connectivity between the hippocampus and PAG [66]. Furthermore, acupuncture treatment can modulate the connectivity of the PAG with the reward system [68]. which in turn was found to be affected in PTSD [69]. During resting state, acupuncture can modulate hippocampal activity according to the selected acupoints. For instance, LI4 and ST 36 produced deactivation in the nucleus-accumbens, amygdala and hippocampus [43]. and PC6 increased functional connectivity between the hippocampus and other DMN nodes [48]. Furthermore, the increased connectivity between DMN and hippocampus was associated with acupuncture-induced increases in parasympathetic tone and parallel decreases in sympathetic tone [48]. Taken together, the modifiable effects of acupuncture on the hippocampus and considering its role in binding event elements, including context and pain, as well as its functional connections with other limbic regions and the DMN, places the hippocampus as a suitable target for PTSD treatment.

#### Acupuncture effects on the DMN

A large portion of brain research on acupuncture focused on the DMN (Default-Mode Network). A prominent feature of this network, which has been the focus of interest in brain research for some time, is that it comprises areas showing decreased activation during tasks that require external attention (goal-directed behavior) as well as increased activity at rest, in the absence of an external task [65]. Studies have shown that this network is associated with not only resting state but also with other cognitive functions, such as autobiographical memory retrieval, active imagining of the future, and delving into the others’ point of view. The prevailing understanding is that the action of the DMN network is the result of collaboration and integration of sub-networks or areas that apparently share a strong affinity for the autobiographical self and the associated memories as an end in itself, as a means of understanding others or as a tool for future planning. As such, the DMN is also associated with pathologies and mental disorders in which these abilities are impaired, such as autism, schizophrenia, and Alzheimer’s disease [70]. The ample research on DMN implies that it is of critical importance for personality features and cognitive functions related to self-definition and self-perception. Furthermore, research shows the importance of the DMN in pain management as part of a dynamic system called the pain connectome, such that mental distraction from the pain leads to an increase in cerebral connectivity in the DMN [71]. Resting-state functional connectivity in PTSD patients shows reduced functional connectivity in the DMN [49]. A recent machine-learning study aimed at mapping PTSD symptoms to brain networks revealed that decreased functional connectivity within the DMN is specifically associated with intrusion and avoidance symptoms in PTSD patients [72].

Acupuncture also affects the DMN. A study at the Martinos Center for Brain Imaging in Boston that examined the effect of true versus sham acupuncture points found that acupoint PC6 can increase brain connectivity in the DMN, in addition to improving connectivity in the ACC, PAG, amygdala, hippocampus, and MTG (middle temporal gyrus). The same study also revealed an increase in parasympathetic activity, along with a decrease in sympathetic activity parallel to PC6 puncturing [48]. Additional fMRI studies have shown that acupuncture points such as ST36, LI4 [73], GB37, KID8 and BL60 74] result in modulation of the DMN. Well-known points have also been associated with general stabilization (ST36) and pain relief (LI4, BL60). Another study revealed a correlation between the intensity of acupuncture manipulation and the intensity of the effect on the DMN [74]. Moreover, there is a high general correlation between acupuncture-associated brain regions and the DMN, to the point that some consider the DMN to be the central brain infrastructure in acupuncture’s effect on the brain [75, 76]. In conclusion, increasing connectivity in the DMN may yield an increase in pain management and a decrease in the PTSD symptoms of avoidance and intrusion. Indeed, the effect of acupuncture in enhancing DMN functional connectivity implies a potentially positive effect of acupuncture on PTSD. The connection between autobiographical memory on the one hand and pain on the other, as emerging from DMN functional connectivity research, together with the influence of acupuncture on the DMN imply that acupuncture is highly relevant to post-trauma, as PTSD inherently binds together memory and pain, whether physiological, mental or both.

## A proposal for an integrated therapeutic approach—three levels of influence

As noted, acupuncture has an impact on three hierarchical levels: a) general sympathetic-parasympathetic balance; b) the amygdala and the limbic system; and c) cortical functional connectivity in central networks, specifically the DMN. All three also correspond with pain-related and memory-related aspects.

### Memory-directed acupuncture (MDA)

**Fig. 1:**
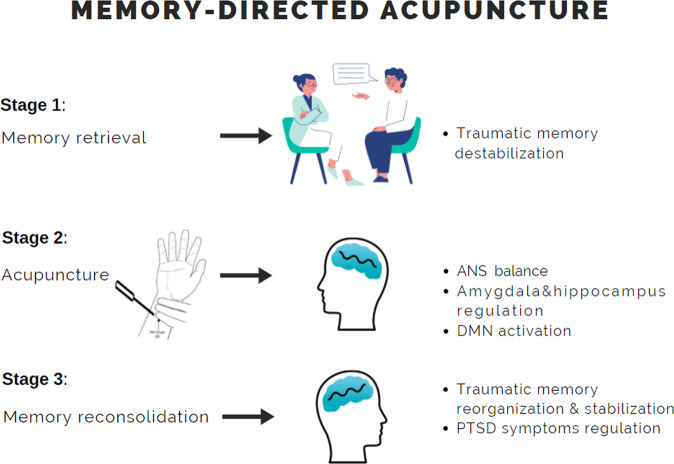
A clinical model for memory-directed acupuncture. Stage 1 includes a therapeutic conversation between the therapist and the patient, in which the traumatic event is retrieved. Following this, in stage 2, an acupuncture session is applied. During stage 3, the targeted traumatic memory that has been evoked (stage 1) is reintegrated through the widespread effects of acupuncture.

Acupuncture is highly relevant for PTSD based upon its ability to reduce hyperarousal, induce general relaxation, and reduce inflammation and symptoms such as sleep deprivation. Yet to date not much research (if any) has been devoted to the effect that acupuncture may have on the traumatic memories, which constitute the main feature differentiating PTSD from other pathologies. The proposed model (Fig. 1) is based on integrating acupuncture’s three levels of influence with memory’s unique ability to change after being retrieved. The therapeutic approach outlined below relies upon these attributes of acupuncture and memory. The approach begins with a therapeutic conversation in which the traumatic event is retrieved, accompanied by all the clinical implications of traumatic memory retrieval. After the relevant memories are evoked, the practitioner applies acupuncture that encompasses the three levels of influence. Thus, the physical intervention of acupuncture along with the preceding conversation can bridge the gap between the mild phase of the clinical conversation and the bodily anchoring of the traumatic event, so that the psychotherapeutic process becomes bodily embedded by targeting the physiological effect of acupuncture into the relevant memories. The acupoints should be delineated by the clinical diagnosis according to the Chinese Medicine theory, clinical experience, and former scientific research. Some acupoints that potentially meet these requirements were mentioned in the former sections, for instance, PC6, [77] ST36 [37, 58, 59, 78] LIV3 [43, 79]. There are other points that are associated with the amygdala, hippocampus and DMN, and with the parasympathetic nervous system, some of which we mention above but are not detailed here, as this is beyond the scope of this paper. Though acupuncture is common practice in most populations, it may be of some burden to PTSD patients, for whom avoidance is a central obstacle in their way to recovery. A recent novel approach that integrates acupuncture with imagery termed video-guided acupuncture imagery treatment (VGAIT) may help overcome this obstacle by allowing a distal treatment that still preserves the therapeutic effect. This new approach paves a pace for different kinds of clinical settings, which may be easier for PTSD patients to experience [80].

Clinical experience with MDA shows that often only a clue or a fragment of the memory is needed, eliminating the high burden of repeated retrieval. This may be explained by the tagging and capture theory, which is based on animal studies [81] and reconfirmed by human studies [82], suggesting that activation of specific memories can induce diffused activation of adjacent memories. This feedback coincides with animal studies showing that repeated retrieval can strengthen fear memory and exacerbate the disorder [83]. Thus, the minimal retrieval of the traumatic memory needed in order to induce the positive effect of acupuncture according to the MDA model may provide a solution to the call emerging from the field to reduce the burden of traumatic memory retrieval. Extensive efforts have been invested in empowering the effects of psychotherapy using interventions such as the administration of beta-blockers or other psychopharmacological agents to enhance reconsolidation. A novel approach to the treatment of PTSD combines exposure therapy with MDMA administration to create a more relaxed and accepting emotional environment. Research into the effectiveness, safety and long-term benefits of this approach is still in its infancy [84]. This approach resembles the proposed model in that both attempt to bridge the gap between psychotherapy and bodily intervention, one by psychopharmacology and the other by neuromodulation using acupuncture. Indeed, similar interventions with barbiturates were in use over 70 years ago [85]. Nevertheless, the effect of the drugs seems to be narrower, leading to limited effectivity.

Unlike psychotherapy, in which creating a therapeutic environment designed to allow safe retrieval of the traumatic memory precedes the memory retrieval itself, in MDA memory retrieval precedes the physiological manipulation, in this case acupuncture.

To conclude, our proposed clinical therapeutic model includes three components: (a) conversational retrieval of the traumatic event or nearby events; (b) subsequent acupuncture based on empiric acupoints for memory-related regions; (c) closure.

A series of such treatments have the potential to exert a significant influence on the traumatic memory, as demonstrated by the following two clinical case studies. Further research is needed to provide empirical support the model.

## Two clinical case studies

Note that all autobiographical details have been omitted or altered to maintain patient confidentiality.

### Case A (Based on Amir Assouline‘s treatment at a private clinic and working with the trauma team at the psychiatric clinic of Ha'Emek Medical Center)

Based on Amir Assouline’s treatment at a private clinic and working with the trauma team at the psychiatric clinic of Ha'Emek Medical Center. E is a member of the security forces who has been investigating demanding and burdensome cases for many years and developed secondary trauma as a result of the cases he investigated. Even though his trauma was “secondary”, he experienced severe post-trauma that included sleep disturbances, harsh intrusions accompanied by tremendous suffering, hyperarousal with constant trembling, outbursts of anger, and deteriorated relationships with loved ones. He also had an episode of major depression and two past suicide attempts. Before acupuncture treatment was initiated, he was treated for about two years at the psychiatric outpatient trauma unit with a combination of antidepressants and psychotherapy. Since the outset of his PTSD symptoms, he exhibited significant fluctuations in functioning. His daily life was characterized by significant avoidance. He was very anxious about his family, especially his children, and prevented them from going to classes or to visit friends.

During the first acupuncture treatment, we established a good rapport and talked freely about why he had seaked treatment and what had caused his condition. The challenging cases he handled remained with him and prevented him from functioning properly, as almost everywhere he went he was flooded by scenes from the past. Based on the Chinese medicine diagnosis, at our next session I applied acupuncture treatment at points KID5, SP4, HT7, PC7, and PC6. When the PC6 acupoint was inserted, I noted a surprising change in his response, which I noted in the clinical diary: “He flinched and seemed to be looking at what was over my shoulder, so much so that I wanted to turn around. I asked him what happened and if he wanted me to remove the needle. He said yes and I took it out. He said that he was thrown into his traumatic memories immediately after the needle was inserted, but he immediately relaxed when I pulled out the needle".

This session took place earlier in the week. The clinical social worker who met him later that same week reported at the staff meeting that he exhibited changes after the session, in particular a very significant reduction in avoidance, even though he seemed to be unaware of these changes. When she drew his attention to this, he also seemed surprised. She linked the surprising improvement to the acupuncture treatment, since prior to that treatment there had been no improvement for quite some time.

The next session also began with a conversation, followed by the same acupuncture treatment. When we got to PC6, I saw he became a bit frightened and alarmed. I reiterated that he is free to stop the treatment whenever he chooses. He tolerated the needle for a few seconds and was again “thrown” into the same memories, followed by a general sense of relief and calm, a common reaction to acupuncture. The behavioral benefits following the treatment continued.

In the third session, I repeated the procedure. When we got to PC6, he was less apprehensive. After the needle was inserted at the point, he again showed an emotional reaction but not a negative one. He reported feeling that “something is happening” in the area of the heart, a kind of “release”. I asked him to focus on his feelings and sensations and try to describe them to me. He described “black tar flowing downwards” (a description repeated by other patients with some variations) and a feeling of release and relief. He showed similar progress in the fourth session, in parallel with behavioral improvement. In subsequent sessions, he was overcome by a sense of calm that he later described as “a calm he had not felt since childhood”. At the same time, his progress continued and his avoidant behavior gradually diminished significantly. When the short series of acupuncture sessions ended, he stated: “I’m fine now”. He continued meeting with the clinical social worker but his condition was much improved.

### Thoughts regarding the treatment

When I saw the reaction on E’s face the first time I inserted the needle at PC6, I realized that something was happening. I thought that perhaps the body’s electrical conduction system might explain the speed and specificity of his reaction to PC6.

The conversation preceding the acupuncture is a major feature of our model. Insights I gained from another patient about two years after E’s case led me to understand that the proximity between acupuncture and the preceding conversation is key to the model’s success. The preliminary conversation, which naturally corresponds with the traumatic events or related issues the patient is dealing with, suggests the possibility that selective stimulation of traumatic memory-related brain areas under positive and controlled circumstances may explain the clinical results. The brain “does the work for us,” bringing us to the relevant region even though only related memories/words/topics were used as stimuli. Experience has taught me that quite a few patients discontinue conversational psychological therapy because of the great difficulty involved in engaging in the traumatic experience and then returning to their everyday lives. They feel that the treatment that is supposed to help them actually pulls them back into the trauma. The ability to “touch” the traumatic memory without consciously retrieving it, as offered in our model (MDA), provides these patients relief without impairing the treatment. As noted, the tagging and capture theory offers a physiological explanation for this. The theory posits that adjacent memories evoke one other, such that the traumatic memory area can be activated without the patient having to recollect a negative experience at the declarative level. E’s case was a milestone for me in that it demonstrated the strong physical involvement and speed of reaction, pointing to significant brain involvement.

### Case B (from the private clinic)

Over a period of three years, M, formerly a soldier in an undercover unit, had no declarative memory regarding his service in the unit. He experienced dissociative amnesia in the sense that he could not remember events or even recognize people from that period. After more than ten years of service in the police, during which he apparently refrained from thinking about that period and from maintaining any contact with anything that reminded him of it, he was invited to a unit reunion and decided to attend. His “fall” came a few weeks later: He began experiencing very strong physical pains, especially in his back, to the point of almost losing the ability to walk. At some point he also reported loss of vision, though a medical assessment found nothing.

After meeting with a psychiatrist, M was diagnosed with posttraumatic disorder and referred for psychological treatment in conjunction with medication (SSRIs). But M felt that “delving into his post-trauma during the therapeutic sessions only knocks me down” and terminated the treatment, a common occurrence among other patients as well. M contacted me after attending a lecture I gave on the topic. He allegedly came to treat severe shoulder pain, but from his background story we both knew that was not the real reason for the treatment. After two treatments targeting his shoulder pain, I told him that in my opinion his post-trauma was his real problem. I proposed treating this problem using the model: a preliminary conversation on the subject of trauma (in this case, there was no apparent memory to hold on to), followed by acupuncture. I explained the general rationale of the treatment.

Neither the preliminary conversation nor the acupuncture treatment was simple. Sometimes we touched upon painful points both in the conversation and during the acupuncture. His complex responses ranged from moments of relief to feelings of severe dissociation and even a kind of paralysis manifested in an inability to move certain organs. Some sessions were discontinued in the middle, seemingly because of his incapacity to bear the unseen burden of the hidden but almost palpable traumatic memory. Nevertheless, M persisted with the treatment. We both noticed that as the difficulty of the preliminary conversation and the acupuncture increased, so too did the subsequent improvement. While I took some liberty in choosing the points, the acupuncture was mostly based on the “prescription” I determined based on theory and on my clinical experience, in which PC6 is a key component. Note that from the moment I began treating the post-trauma according to the model, M’s physical pain almost immediately disappeared, and his behavior also began showing a great deal of progress. Until the treatment, M’s sleep was marked by nightmares, from which he would wake up sweating but unable to remember his dreams at all. Already in the early stages of treatment, his sleep and dreams returned to normal and he gradually stopped taking sleeping pills.

His daily functioning improved to the point that he asked for a meeting with the psychologist at the government agency where he worked to obtain permission to return to his former prestigious position, from which he had been removed following the psychiatric diagnosis. At first, the psychologist was very skeptical although very surprised by the change in M. Eventually he was given the opportunity to return to his former position. M’s wife also testified to the significant change in him. Although the memories of his military service still remain largely hidden, he seems able to remember more details from those years. M was aware of his progress and wrote a letter describing the changes he experienced and illuminating the key points in the treatment. Like E, M has arrived at a point where he can say: “I’m fine today and I do not want to risk reopening that memory”. In terms of clinical diagnosis, M reached full remission, even though he admits he “will always carry the scars of the trauma”.

E and M both regained the ability to function emotionally, professionally and in the family. They both arrived at a point where they are satisfied with their lives. To the best of my knowledge, their improvement has continued at least two years beyond their treatment.

## Discussion and summary

This paper describes a new paradigm for treating PTSD based on Chinese medicine, which is backed by 3000 years of medical tradition but has also been scientifically researched for the past 50 years. The paradigm relies on a well-established memory model embedded within current neurobiological research and is based on cumulative clinical experience over the past decade. The paper describes two cases within the limits of therapeutic confidentiality.

In his book *The Body Keeps the Score* [2], Van der Kolk outlines three main avenues for dealing with post-trauma: (1) conversation, which entails talking to others and allowing ourselves to understand what is happening to us while processing traumatic memories; (2) turning off hyper-reactions by medications or other techniques that change the way the brain organizes information; (3) allowing the body to have experiences that on a deep and internal level constitute the opposite of the helplessness, anger, and collapse resulting from the trauma. He concludes by stating that often the best way is to apply these avenues together [2].

The model proposed in this paper adopts the three channels suggested by van der Kolk, linked together into one therapeutic method, as evident in the structure of the treatment and its underlying rationale. The therapeutic session opens with a conversation about the traumatic memory and its various aspects (related memories can also be as helpful) and about the patient’s daily routine and personal and family life. The therapist should be sensitive enough to elicit the appropriate degree of openness in the conversation, but not beyond this as exaggeration will generate an emotional flood that may delay or even prevent further treatment. At this point, the therapist can give the patient tools for daily coping and work on bringing the traumatic event into the appropriate framework or context within the autobiographical memory sequence. This stage corresponds with Van der Kolk’s first stage and is also preparation for the next stage that includes acupuncture.

The activation in the conversation sets the stage for the neuromodulation effect of acupuncture on the traumatic memory-related area of the cortex, parallel to the systematic regulation induced by the acupuncture. Complete retrieval of the traumatic memory can sometimes impair the therapeutic process. In MDA the traumatic memory should not always be fully retrieved and thus can be mitigated by partial retrieval, as the neuromodulation that yielded from acupuncture may be targeted towards the themes and memories that were already aroused in the preceding conversation.

In MDA, memory processing takes on another dimension that is beyond the conversation and is anchored in the body, in the flesh. Acupuncture affects various physiological layers, beginning with the sensory system, continuing to the autonomic nervous system, via regulation of the sympathetic-parasympathetic tone, mediated by the HPA axis, and ending in the limbic system and important neural networks in the cerebral cortex. In one of my first treatments based on the model, the patient told me he felt a calmness he “has not felt since childhood.” I told him he should remember this feeling and realize that it still exists somewhere and he is still able to feel it. This parallels the third avenue described by Van der Kolk in that it creates another physical experience contrary to trauma-derived helplessness, anger, and collapse.

Nevertheless, it should be noted that memory-directed acupuncture is still based on anecdotal evidence and requires extensive research to establish its clinical validity and safety. One goal of this article is to recruit the resources needed for this broad clinical research. We believe that after a long period of successful application of MDA in the clinic, the time has come for randomized controlled trials to explore its validity so that it can be applied more widely and offer healing possibilities to more individuals whose disease has been defined by many as incurable.
